# Design and Optimisation of Sustainable Sample Treatments Based on Ultrasound-Assisted Extraction and Strong Cation-Exchange Purification with Functionalised SBA-15 for Opium Alkaloids in Ground Poppy Seeds

**DOI:** 10.3390/toxins15120672

**Published:** 2023-11-24

**Authors:** Gema Casado-Hidalgo, Sonia Morante-Zarcero, Damián Pérez-Quintanilla, Isabel Sierra

**Affiliations:** Departamento de Tecnología Química y Ambiental, E.S.C.E.T, Universidad Rey Juan Carlos, C/Tulipán s/n, Móstoles, 28933 Madrid, Spain; gema.casado@urjc.es (G.C.-H.); sonia.morante@urjc.es (S.M.-Z.); damian.perez@urjc.es (D.P.-Q.)

**Keywords:** opium alkaloids, ground poppy seeds, ultrasound-assisted extraction, solid-phase extraction, SBA-15 functionalised with sulfonic groups, HPLC-MS/MS, food safety, natural toxins

## Abstract

An analysis methodology was optimised and validated for the quantification of opium alkaloids (OAs) in ground poppy seeds. This involved ultrasound-assisted extraction (UAE) and solid-phase extraction (SPE) purification before analysis using a high-performance liquid chromatography mass spectrometry detector (HPLC-MS/MS). UAE was optimised through the design of experiments with three factors and a three-level full factorial design. For SPE optimisation, a commercial material was compared with a previously synthesised material of SBA-15 silica functionalised with sulfonic groups (SBA-15-SO_3_^−^). The synthesised material demonstrated superior efficiency with only 25 mg and proved to be reusable for up to four cycles. The methodology was properly validated in terms of linearity, limits of detection and quantification, and selectivity. Matrix effects were negligible; adequate recovery values (85–100%) and inter-day and intra-day precision (≤15%) were obtained. The greenness of the method was evaluated with the AGREEprep metric scale, being more environmentally friendly compared to OA analysis methods. Finally, the method was applied to different samples of ground poppy seeds and revealed a concentration of 140 mg/kg of morphine equivalents in one of the samples, surpassing the legislatively established limits by sevenfold. This highlights the need to analyse these types of samples to mitigate potential public health issues.

## 1. Introduction

There is a growing trend to incorporate seeds into food for both sensory enjoyment and nutritional benefits, such as poppy seeds (from *Papaver somniferum* L.) [1]. These seeds are widely consumed in Central Europe, and their use is increasingly prevalent across various food products. Notably, poppy seeds are used in bakery products (including different types of bread or biscuits), as well as in yoghurt and even for preparing infusions [2]. Additionally, poppy seeds are commonly ground and employed as fillings in traditional sweet dishes and pastries, such as cakes, strudels, fritters, pastries, or poppy dumplings [2,3]. Nevertheless, it is crucial to be aware that poppy seeds have the potential to be contaminated with opium alkaloids (OAs) when they come into contact with the latex of the plant, which is rich in OAs. Significantly elevated concentrations, possibly attributed to automatic harvesting methods or insect damage, have been observed [4]. The consumption of contaminated poppy seeds poses risks, including intoxication and positive results in drug tests for athletes or workers [5]. Consequently, a maximum limit of morphine equivalents (morphine + 0.2 × codeine) in poppy seeds has been established [6]. Nevertheless, this legislation only involves two OAs (morphine and codeine). Given findings from previous studies indicating the presence of other OAs (such as thebaine, papaverine, noscapine, and oripavine) in substantial concentrations, health authorities emphasise the need for further research to control these additional OAs, which may possess higher toxicity than morphine or codeine [5,7]. Presently, there is a lack of a properly validated method specifically designed for the analysis of ground seeds; existing methodologies are tailored only for whole seeds. This is a critical consideration, as ground seeds are widely used and are more complex samples. In addition, it is important to highlight that grinding has been shown to decrease the morphine content in seeds by approximately 25–34%, as is shown in an EFSA recommendation and in other studies [8,9]. However, further studies are required to determine the grinding conditions leading to degradation and to assess the influence of other OAs [10]. Therefore, to study all this, there is a pressing need to develop a rapid, efficient, and environmentally friendly analytical method for quantifying all six OAs in ground poppy seeds.

For this purpose, sample preparation is a key step in the analytical method when dealing with analytes present at low concentrations in highly complex matrices [11]. Historically, the primary methods for extracting OAs have relied on liquid-liquid extraction (LLE). However, this approach typically involves the use of high volumes of solvent, long extraction times, and sometimes successive extractions to achieve full recovery. Unfortunately, these aspects make the methodology less environmentally friendly [4]. For these reasons, there is a trend towards the development of more environmentally sustainable methodologies, characterised by shorter extraction times and smaller solvent volumes [12,13]. An emerging and popular technique in this regard is ultrasound-assisted extraction (UAE), known for its increased sustainability. UAE enables high extraction yields with less solvent consumption, attributed to the cavitation effect induced by ultrasound [14,15]. The efficiency of UAE is influenced by numerous variables, including solvent type, solid/liquid ratio, and extraction time, emphasising the importance of optimisation to maximise extraction efficiency. Response surface methodology (RSM) proves highly valuable in this context, allowing for the systematic optimisation and evaluation of multiple factors at different levels and their possible interactions. RSM is based on fitting a polynomial model equation to various experiments, describing the characteristics of the dataset, and additionally providing statistical prediction equations [16,17]. This extraction technique has previously demonstrated success in extracting OAs from bakery products [18].

On the flip side, achieving an efficient extraction of OAs from the matrix poses a challenge, given that ground poppy seeds are complex samples with many components characterized by diverse physico-chemical properties. Consequently, a crucial step in the process involves purification to eliminate potential interferents from the extract, ensuring a cleaner extract and preventing contamination that may harm the equipment’s column or the detector. The widely adopted method for this purpose is solid-phase extraction (SPE), chosen for its notable advantages [19,20]. In previous studies, a variety of commercial sorbent materials have been employed for clean-up purposes. Examples include the use of the Chem Elute column to purify morphine and codeine in serum and urine after ingestion of poppy seeds [21], Clean Screen ^®^ DAU to purify morphine in human urine [22], and Oasis ^®^ MCX and Oasis ^®^ HLB to purify five alkaloids of *Pericarpium Papaveris* in a hot pot [23]. However, a current trend in analytical chemistry involves the synthesis and development of new absorbent materials with more controlled and improved textural characteristics, allowing stronger and more specific interactions with target analytes [20,23,24]. Mesostructured silicas, such as SBA-15 (Santa Barbara Amorphous-15), have garnered increasing interest in sample preparation research [18]. This is attributed to its fast, cost-effective, and straightforward synthesis, yielding a sorbent with many advantages. These include a highly ordered and size-controlled structure, a high surface area, a large pore volume, good chemical stability, and the ability to be functionalised with different functional groups [25]. Notably, these functional groups contribute to active sites that are more selective than pristine SBA-15. For instance, strong ion-exchangers as sulfonic acids, in addition to providing hydrogen bonds through the free silanol groups of the silica, induce retention towards cationic compounds due to the presence of anionic functionalities (SO_3_^−^) [26]. The described interactions provide greater strength and selectivity, facilitating a reduction in the required quantity of material. This not only enhances efficiency but also aligns with environmentally friendly practices.

Therefore, the aim of this study was to develop and validate an efficient and environmentally friendly analytical method for quantifying six OAs in ground poppy seed samples. This involved using a UAE-SPE sample preparation protocol with a strong cation-exchange purification step using a functionalised SBA-15 silica with sulfonic groups (SBA-15-SO_3_^−^) as the solid phase, followed by analysis using liquid chromatography coupled to a triple quadrupole tandem mass detector (HPLC-MS/MS). To obtain the highest recovery values for a faster, simpler, and more environmentally friendly methodology, the extraction conditions in the UAE were optimised using response surface methodology (RSM).

## 2. Results and Discussion

### 2.1. Characterisation of the Synthesised Material

To verify the successful synthesis, elemental analysis (EA) was performed to estimate the degree of functionalisation of the material. Nitrogen gas adsorption–desorption isotherms were also employed to evaluate the pore volume, surface area, and pore distribution (for more details, see Supplementary Information S1). First, the EA was carried out for both the final material with the SO_3_^−^ groups and the non-oxidised material with the (SH^−^) groups. For the non-oxidised material, the C and S ratios were 4.378 and 1.473%, respectively. In contract, for the final oxidised material, these ratios decreased to 4.042 and 1.344%, respectively. The decrease in both percentages after oxidation validates the correct oxidation and functionalisation of the material. In addition, the quantity of ligand in the final material was estimated at 1.19 mmol S/g of material, aligning with values obtained by our research group [26]. The specific surface area (S_BET_) of the material was 563 m^2^/g, with an average pore volume of 0.67 cm^3^/g and an average pore diameter of 49.2 Å. These results coincide with expectations, according to previous results obtained in our research group [26].

### 2.2. Optimisation of the Sample Preparation

#### 2.2.1. Optimisation of the Purification via SPE

**Figure 1:**
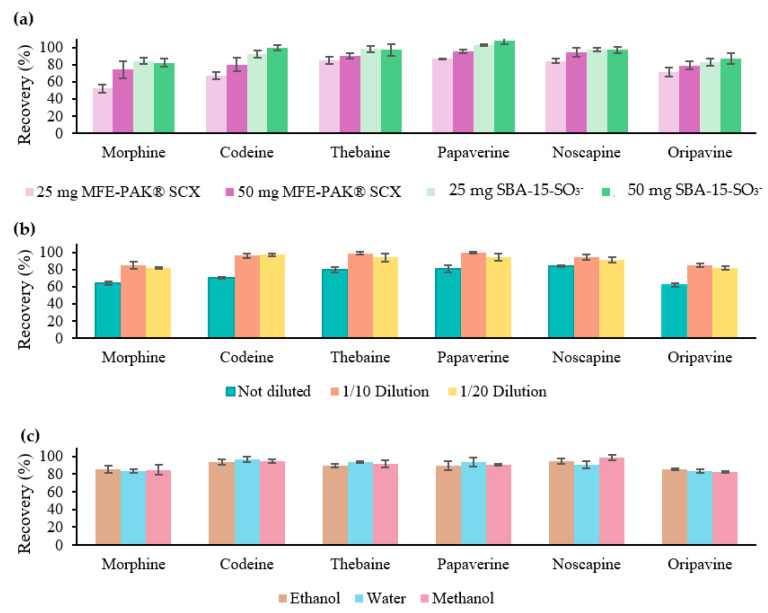
Recovery values (%) obtained with 25 and 50 mg of MFE-PAK^®^ SCX and SBA-15-SO_3_^−^ material at the same SPE conditions with standards (**a**). Recovery values (%) obtained with the sample extract not diluted, 1/10 dilution, and 1/20 dilution with 25 mg of SBA-15-SO_3_^−^ (**b**). Recovery values (%) obtained with the sample extract diluted in ethanol, water, or methanol 1/10 in water with 1% HCl with SBA-15-SO_3_^−^ (**c**).

First, a comparison was conducted between the synthesised material (SBA-15-SO_3_^−^) and a commercial material (MFE-PAK^®^ SCX) using two different amounts of material (25 and 50 mg). Both materials were subject to the same conditions of conditioning (2 mL of water with 1% HCl), loading (2 mL of 0.5 mg/L OAs in water with 1% HCl), and elution (2 mL of methanol with 5% ammonia). The results, as shown in Figure 1a, reveal favourable recovery values (%) with both materials with 50 mg of material were favourable for all analytes: 82–100% in the case of SBA-15-SO_3_^−^ and 72–96% with MFE-PAK^®^ SCX. Notably, with only 25 mg of material, the synthesised material achieved significantly higher recovery values compared to the commercial material (92–103% vs. 52–83%). Therefore, the synthesised material showed enhanced efficiency, as recovery values did not decrease with lower material amounts. Therefore, 25 mg of SBA-15-SO_3_^−^ was selected for the present work. These results can be attributed to the higher S_BET_ of the SBA-15-SO_3_^−^ material compared to the MFE-PAK^®^ SCX sorbent (563 and 398 m^2^/g, respectively) and a higher functionalisation degree (1.19 and 0.8 mmol SO_3_^−^/g, respectively). Therefore, the SBA-15-SO_3_^−^ material has larger active sites for interaction, resulting in a higher efficiency than the commercial sorbent.

After confirming the efficacy of the synthesised material and optimising the required amount, a re-optimisation of the procedure was carried out. This consisted of determining whether 1% ammonia in methanol (instead of 5%) was sufficient to obtain adequate recovery values. The objective was to determine if this adjustment could enable the injection of purified extracts directly into the equipment without the need for evaporation, thus considerably shortening the analysis time. The results indicated that there were no significant differences in the recovery values obtained when using 1% and 5% ammonia. Consequently, the 1% ammonia solution was selected for subsequent steps.

Following the optimisation of the SPE procedure with standards, the next step involved optimising the procedure using the extract obtained from the sample. First, the recovery values corresponding only to the SPE procedure were evaluated after passing 2 mL of the sample undiluted, as well as extracts diluted 1/10 with water containing 1% HCl and diluted 1/20. To carry out this determination, a sample extract was taken and spiked just before the purification process at a known concentration (1 mg/L). The ground poppy seed sample extract was prepared using the initial UAE conditions (0.5 g sample with 10 mL of 1% HCl water for 10 min of ultrasound). The recovery values are shown in Figure 1b, whereas acceptable results were achieved with dilutions. Consequently, it was concluded that a 1/10 dilution of the extract at the loading stage of the SPE procedure was necessary.

Subsequently, as the solvents used for the optimisation of the UAE will be water, methanol, and ethanol, the SPE procedure was evaluated as showing adequate recovery values with each solvent. Therefore, three extracts were prepared with the initial conditions of the UAE and each of the three types of solvents. Subsequently, each of the extracts was spiked to a known concentration (1 mg/L), and a 1/10 dilution with water with 1% HCl was carried out. As shown in Figure 1c, no significant differences were shown between the three types of solvents used. Adequate recovery values were obtained in all cases. Therefore, it was concluded that the SPE step was successfully optimised, and an evaluation of the UAE step was carried out with the certainty that lower recovery values than those obtained at this point were due to insufficient extraction in the UAE step.

#### 2.2.2. Optimisation of UAE

##### Effects on the Extraction of the Main UAE Variables and Statistical Analysis

The experimental factorial design methodology incorporated a categorical factor, representing the type of solvent (A), and two numerical factors, which are the solid/liquid ratio (B) and the extraction time (C). This approach aimed to investigate how these three independent variables affect the UAE process. Each factor was evaluated at three levels (3^3^) to determine the optimal conditions that yield the maximum recovery values (dependent variable) with the lowest extraction time and solvent volume, thereby enhancing the environmental friendliness of the methodology. Thus, a full factorial design of 27 experiments was established, representing a three-factor full factorial design at the three most promising levels, as determined through preliminary experiments and previous research (Table S1). In addition, based on our previous work [18], experiments were carried out with the UAE at a set amplitude (75%) and pulsed sonication mode (2:1). The recovery values for each of the six analytes served as the dependent variables.

The results from the design of the experiment are summarised in Table 1, showing the mean of the values obtained for each response with three replicates of each study ± standard deviation (SD).

The experimental values for each response varied within the following ranges: 10 ± 2 and 82 ± 1% for morphine, 17 ± 2 and 100 ± 4% for codeine, 24 ± 3 and 99 ± 1% for thebaine, 5 ± 3 and 100 ± 4% for papaverine, 18 ± 1 and 100 ± 5% for noscapine, and 26 ± 1 and 82 ± 4% for oripavine.

**Figure 2:**
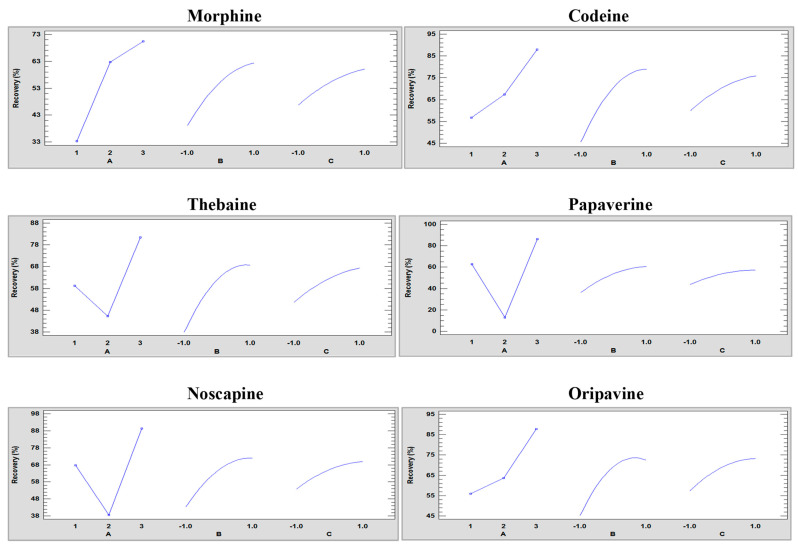
Main effect plots of the full factorial design 3^3^ of each of the responses showing the three factors at three levels: (A) solvent type (1: ethanol, 2: water, and 3: methanol), (B) solid/liquid ratio (−1: 0.5 g/3 mL, 0: 0.5 g/5 mL, and 1: 0.5 g/10 mL), and (C) extraction time (−1: 3, 0: 5, and 1: 10 min) to UAE optimisation.

In addition, to evaluate the different types of effects of the variables, graphs were plotted, illustrating each of the main effects of each variable. This graphical representation aids in determining the positive or negative effect of each variable, as shown in Figure 2.

The solvent with the highest recovery values was methanol for all analytes. Distinct behaviours were observed between ethanol and water, depending on the analytes. Morphine, codeine, and oripavine exhibited higher recovery values with water, while thebaine, papaverine, and noscapine showed higher recovery values with ethanol. This variation can be attributed to the nature of each compound, as the more polar ones were better extracted with water and the more non-polar ones with ethanol. However, the recovery values were only favourable for all analytes with methanol, which has an intermediate polarity. On the other hand, the solid/liquid ratio displayed a positive effect on recovery values. However, as shown in Figure 2, there is no linearly increasing trend, and the maximum recovery value did not correspond to the highest solid/liquid ratio studied. A plateau is observed in the final part, which may indicate that there was an intermediate optimum value at these levels. The same occurs when the extraction time increases, and the longer the extraction time, the higher the recovery values.

A statistical analysis (ANOVA) was carried out, and the obtained statistical parameters (R^2^, Adj.; R^2^, Pred.; and R^2^ and *p*-values) were obtained as shown in Table S2. All statistical parameters indicated that the quadratic models exhibited very high predictability, as most coefficients were close to 1. In addition, ANOVA confirmed the variables that showed statistically significant differences in the quadratic models, where values of p were lower or equal to 0.05. For the recovery of codeine, papaverine, and noscapine, the statistically significant individual variables were solvent type (A) and solid/liquid ratio (B). In the case of the recovery of morphine and oripavine, besides those two, it was also the extraction time (C). Thebaine recovery only showed differences with the solid/liquid ratio. In terms of the combinations among the variables, the ones that presented statistically significant differences were the quadratic value of the solid/liquid ratio for all analytes. For the recovery of papaverine and noscapine, it was also the combination of solvent type and solid/liquid ratio (AB). Finally, for the recovery of morphine and oripavine, the quadratic value of the extraction time (CC) also showed statistically significant differences.

##### Optimisation of the Most Influential Variables of the UAE through RSM Approach

**Figure 3:**
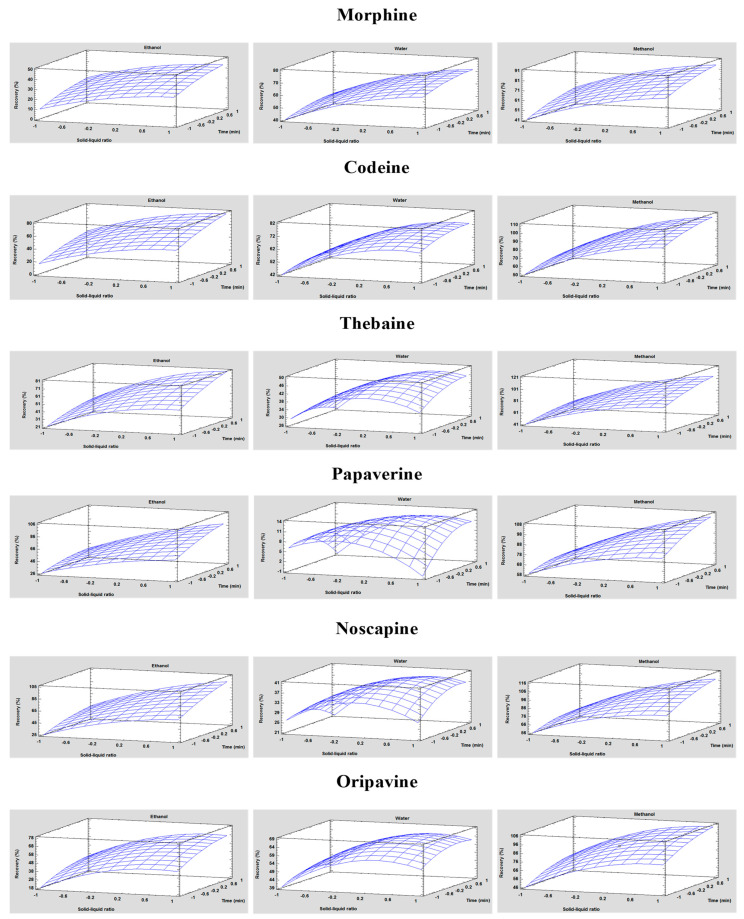
Plots of the response surface demonstrate the effects of the solid/liquid ratio and the extraction time with each of the three solvent types on the recovery of each analyte for UAE optimisation.

After establishing that all the independent variables and certain combinations showed statistically significant differences in responses, a multiple regression analysis was conducted to obtain mathematical models, as represented in Table S3. Table S3 effectively elucidates the empirical relationship among the three studied variables and various responses. In addition, Figure 3 displays the relationship between dependent and independent variables through response surface plots generated from the acquired polynomial equations. Notably, for the three types of solvents, the recoveries increased with increasing solid/liquid ratios in the same way as they increased with increasing extraction time. However, in the case of water, a point was reached where both the solid/liquid ratio and extraction time no longer contributed to higher recovery values, indicating a plateau in the graph. In contrast, for methanol, the graph shows a much more pronounced increase. This is because the highest recovery values for all analytes were obtained with this solvent.

To determine the optimum level of each of the independent variables, numerical optimisations were carried out. For all analytes, a similar value was obtained, between 7.55 and 8.5 mL of solvent volume and 4.12 and 5.5 min of extraction time. Consequently, the selected optimal conditions were 8.5 mL of solvent volume for 5.5 min of extraction time for the optimal recovery of each analyte.

The experimentally determined results were compared with the results predicted using the mathematical equations derived from the RSM models. The aim was to assess the reliability of the RSM for quantitative prediction. The obtained values were very similar, confirming the effectiveness and validity of the response surface models.

### 2.3. Method Validation

The validation results of the optimised analytical method for the quantification of six OAs in ground poppy seeds are compiled in Table 2. The linear range for most analytes started at 0.001, except for oripavine, which began at 0.005 mg/L. The upper limit for all analytes was 1 mg/L. Additionally, calibration lines showed adequate R^2^ values, ranging between 0.994 and 0.996. In addition, slope deviations were calculated for three different days (n = 3) to ensure reproducibility. Low RSDs between 0.3 and 10.9% were obtained, demonstrating good linearity. In addition, the deviations of the back-calculated concentrations of the calibration standards from the real concentrations in the matrix calibration lines were also calculated, yielding adequate values (≤±20%), specifically falling between −1.2 and 17.3% [27].

To evaluate the possible matrix effect that may remain after doing the purification step, the slope of matrix-matched calibration curves was divided by solvent-based calibration curves. The results, presented in Table 2, ranged between −11 and 9%. In this way, as indicated in the validation guide, the matrix effect is less when it is closer to 0%, and the matrix effect is considered negligible when it is less than ±20%. If the result is more than 20%, this indicates enhancement of the signal, and results below 20% indicate suppression of the signal. In addition, it should be noted that if the signal suppression or enhancement is greater than this 20% margin, matrix effects must be taken into account in the calibration [27]. Therefore, this may mean that the procedure of purification developed was able to eliminate almost all possible matrix effects for all OAs.

To calculate method detection limit (MDL) and method quantification limit (MQL) values used, the lowest concentration was analysed and estimated as the lowest concentration, giving a signal-to-noise ratio (S/N) of 3 or 10, respectively. The obtained results were low enough to quantify the samples, specifically 0.007 and 0.03 mg/kg for noscapine, 0.01 and 0.05 mg/kg for thebaine and papaverine, 0.03 and 0.1 mg/kg for codeine, 0.06 and 0.20 mg/kg for morphine, and 0.2 and 0.5 mg/kg for oripavine, respectively.

Concerning accuracy and precision, both were evaluated at three different concentration levels, specifically a high level of 40 mg/kg, a medium level of 20 mg/kg, and a low level of 3.4 mg/kg. The results indicate satisfactory recovery values between 85 and 100% (Table 2). Additionally, intra-day and inter-day precision were evaluated at these three concentration levels. The RSD values were all below 15%, demonstrating adequate precision according to established guidelines [27].

Furthermore, a good selectivity of the analytical methodology was obtained because the variation of the retention time was ≤0.1 min and the ion ratios were within ± 30% (relative abundance) between the sample extracts and the mean of the standards for each analyte [27].

### 2.4. Evaluation of Material Reuse

The potential for material reuse is a crucial aspect to consider in the methodology. Reusing the material not only enhances the economic feasibility of the method but also contributes to its environmental friendliness.

**Figure 4:**
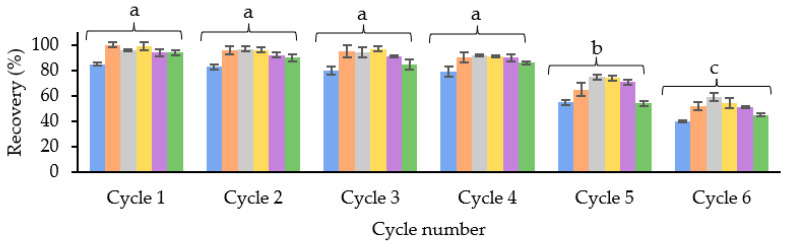
Average recovery values (%) (*n* = 3) obtained in each of the reuse cycles of the SPE cartridge with 25 mg of SBA-15-SO_3_^−^ material. Different letters mean significant differences if *p* ≤ 0.05.

To evaluate this, a study was carried out with three different cartridges (n = 3) with a blank sample extract and spiking it before the SPE procedure with 0.5 mg/L of OAs. Six cycles were run to evaluate at which point the recovery values started to decrease. Between the cycles, the cartridge was washed to remove any retained matrix interference. For this, 2 mL of the elution solvent and 2 mL of the conditioning solvent were passed through, drying for 1 min between the two steps. As shown in Figure 4, the recovery values started to decrease considerably in cycle 5. Therefore, it was concluded that the cartridge could be reused four times without losing efficiency.

### 2.5. Comparison to Other Methods and Greenness Evaluation

It is noteworthy that, as of now, there is no optimised and validated methodology specifically for ground poppy seeds, which present a more complex matrix compared to whole seeds. Therefore, the analytical methodology of the proposed method was compared to previous methods for the determination of OAs in unground poppy seeds, such as shown in Table S4.

It should also be noted that the previously published works in this field often do not incorporate a purification or cleaning step in their methodologies. The absence of such a step can lead to extracts from complex matrices containing large amounts of matrix interferents. This may result in extracts that are more turbid and dirty, potentially causing damage to the chromatographic column and the ionisation source in the analytical instrument [28]. On the other hand, in other works such as Sproll et al. (2006) [29] and Carlin et al. (2020) [30], neither the matrix effect nor the recovery values of the method have been studied, which shows an incomplete validation of these methodologies. On the other hand, our research group developed a methodology for the analysis of OAs in seeds [4] and obtained a slight matrix suppression effect in some analytes and an increase in others. In the same way, recovery values slightly below 70% and above 100% were obtained. Therefore, it can be stated that the methodology proposed in the present work is the most suitable for the analysis of OAs in ground poppy seeds of those published to date.

**Figure 5:**
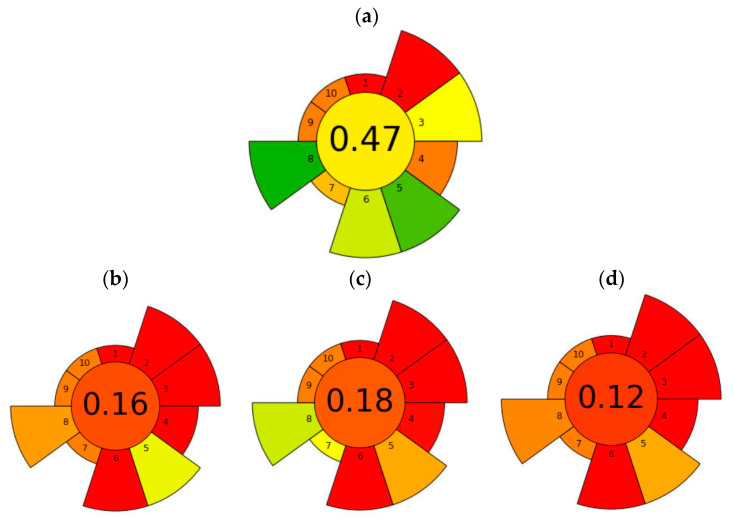
Evaluation of the greener profile of the proposed methodology in this work based on UAE-SPE-LC-MS/MS (**a**), a method based on SLE-MSPE-LC-MS/MS [4] (**b**), a method based on SLE-LC-MS/MS without the purification step [29] (**c**), and another method based on SLE-LC-MS/MS [32] (**d**) using the AGREEprep metric proposed by Pena-Pereira, Tobiszewski, Wojnowski, and Psillakis [31].

On the other hand, when comparing methods, it is also important to consider the environmental impact and safety aspects, often referred to as “greenness”. To evaluate these factors, an assessment of greenness was performed based on the recently introduced metric tool AGREEprep [31]. The method proposed in the present work obtained the highest overall score of 0.47 (Figure 5a). Regarding the highest scoring items, the #2 hazardous solvents and reagents were the lowest scoring items due to the use of MeOH for both extraction and elution of the SPE (Table S5). However, the item #Target—sustainable, reusable, and renewable materials—was more favourable and was considered to have the highest score, since in any methodology where a purification step is performed with materials, it is essential that they can be reused and to minimise all costs and residue that can be generated from the performance of a synthesis. In addition, the maximum score was also given to the #minimize sample amount and the #maximize sample throughput that could be performed in one hour, as well as the amount of energy consumed per sample. The greenness assessment result obtained for the proposed methodology was compared with previous analytical methodologies. For instance, a methodology previously optimised to analyse whole seeds relied on SLE and a subsequent purification via magnetic solid-phase extraction (MSPE) [4]. In this case, the score obtained was 0.16 (Figure 5b). This was because the volume of toxic solvent was higher (34 mL), the material used for MSPE could not be reused, the number of seeds used was higher (2.5 g), and the extraction time was longer, namely 1 h of magnetic stirring (Table S5). In addition, the energy required for each sample was high (147.74 Wh) due to the different steps and a solvent evaporation step. Another example in which the number of steps is reduced and no purification is performed despite the matrix effect is the work of Sproll et al. [29]. In this case, the work obtained the highest overall score of 0.18 (Figure 5c). Although it was a methodology that had only one step in sample preparation and did not use much energy, the volume of toxic materials used (30.06 mL) and waste produced was high (50 Wh). In addition, the amount of sample used was higher, and the extraction time was 1 h (Table S5). Another example similar to the previous one is the EURL-MP-method_007 [32], which is a method developed by the EU Reference Laboratory for Mycotoxins & Plant Toxins in Food and Feed (EURL-MP) to determine morphine and codeine in poppy seeds. A highlight of this methodology is that it is the only one previously published in which poppy seeds are ground to determine the concentration of OAs, which according to the EU recommendation in 2014 would not be very convenient, as grinding is a culinary processing that could decrease the morphine content of the seeds by 25–34% [8]. On the other hand, when subjecting the conditions of the methodology to the AGREEprep metric scale, it obtains the lowest score of the evaluated methodologies (0.12 points). This is mainly due to the large volume of solvent used to perform the SLE (100 mL), the high sample size (10 g), and the high extraction times (30 min stirring, 15 min waiting, and 10 min centrifugation) [32].

In conclusion, the optimisation of the sample preparation step is crucial to achieving a greener analytical methodology. It is not always the case that a purification step worsens the greening of the method. As demonstrated in these examples, it has been clearly demonstrated that this is not the case and that it is also very important to remove matrix interferents to obtain cleaner extracts. The most important factors influencing the environmental impact of these types of methods are solvent volume, solvent type, extraction time, and sample amount. Therefore, it is important to optimise SLE as much as possible to use less sample, solvent, and time, and replacing SLE with UAE can help to further decrease these factors, making the analytical methodology more environmentally friendly.

### 2.6. Application of the Proposed Methodology to OA Analysis in Ground Poppy Seeds

The proposed method was successfully applied to the analysis of three different types of ground poppy seeds. The obtained areas for each analyte could have been interpolated in the solvent calibration line directly because the ME in all cases was negligible. In fact, quantifications were checked with both lines after confirming the absence of a matrix effect, and the concentrations obtained were the same. This indicates that the use of an internal standard may not be necessary, potentially reducing additional costs. However, to further validate the reliability of the developed method, internal standards were used to correct the signals of the matrix-adjusted calibration. Morphine-d3 was used to correct the signals of all analytes except codeine, which was corrected with codeine-d3. Table 3 shows the mean concentrations (mg/kg) of each analyte obtained from six replicates (*n* = 6) for each of the three different samples.

In sample GPS-01, the six analytes were detected, but all were at very low concentrations, the highest being morphine and codeine with 0.8 ± 0.2 mg/kg and 0.5 ± 0.1 mg/kg, respectively. In sample GPS-02, concentrations were higher for morphine and noscapine, with 4.2 ± 0.6 mg/kg and 3.7 ± 1.1 mg/kg, respectively. However, these concentrations were below the maximum limit set by legislation of 20 mg/kg morphine equivalent. However, sample GPS-03 showed high levels of OAs, with 134 ± 1.6 mg/kg morphine, 82.1 ± 6.3 oripavine, 75 ± 1 thebaine, 28.2 ± 0.1 codeine, and 0.9 ± 0.1 mg/kg noscapine. This sample showed a morphine equivalent value exceeding the legal limit (140.24 mg/kg) by seven times, posing a significant risk to consumers’ health. This demonstrates the importance of studying these samples to ensure compliance with legislation. In addition, non-legislated analytes also showed significantly high concentrations. Therefore, these analytes should be considered in future studies.

The concentrations obtained in the ground seeds in this study align with those obtained in previous research analysing whole poppy seeds [4,29,30,33]. In all these studies, as in this one, notably high concentrations of OAs were found, which pose a health risk to the consumer. However, in other studies and in accordance with the EFSA recommendation, it is suggested that grinding could reduce the OA content in seeds by 25–34% [8,9]. This reduction could be attributed to their degradation, primarily through oxygen, leading to the formation of other compounds such as n-oxide morphine and pseudomorphine. Given the variability in the concentration of OAs in poppy seeds, further research challenges would be to carry out additional studies to determine the real influence of this type of processing. Furthermore, understanding the possible degradation compounds formed is crucial to ascertaining whether they are even more toxic [10].

## 3. Conclusions

The first analytical methodology for quantifying six OAs in ground poppy seeds has been successfully optimised and validated. Moreover, this methodology stands out for its simplicity, speed, and environmental friendliness compared to similar approaches. This is due to the performance of the UAE, which requires shorter extraction times and lower solvent volumes. In addition, the SBA-15-SO_3_^−^ material showed a high efficiency because only 25 mg of material was needed, and it was possible to reuse it up to four times without losing efficiency. Furthermore, the methodology showed negligible matrix effects, adequate recovery, and precision values, as well as the rest of the validation parameters. Finally, the methodology was used to analyse three ground seeds, and it was shown that one of them contained quantities much higher than those established by the legislation and of the rest of the OAs that are not regulated. This emphasises the imperative to rigorously control the concentrations of OAs in such samples, reinforcing the importance of this developed methodology in ensuring the safety and compliance of products derived from ground poppy seeds.

## 4. Materials and Methods

### 4.1. Reagents and Materials

Standards of thebaine, morphine, oripavine, and codeine were acquired from Alcaliber S.A.U. (Madrid, Spain). Papaverine, noscapine, morphine-d_3_, and codeine-d_3_, such as internal standards (IS), were purchased from Sigma-Aldrich (Zwijndrecht, The Netherlands). Stock of each of the standard solutions was prepared at 1000 µg/mL in methanol, and working standard solutions were prepared by mixing each of the target analytes at 1 µg/mL in methanol with 0.1% formic acid. These solutions were stored at −20 °C in darkness.

Hydrogen peroxide (H_2_O_2_, 30%) and hydrochloric acid (HCl, 37%) were acquired from Scharlab (Barcelona, Spain). 3-mercaptopropyl triethoxysilane (MPTES, 94%) was obtained from Alfa Aesar (Karlsruhe, Germany). Tetraethylorthosilicate (TEOS, 98% CAS 78-10-4, MW = 208.33 g/mol) and ethylene glycol (poly(ethylene glycol)-block-poly) (EO20PO70EO20, Pluronic^®^ 123, P123, MW = 5800 g/mol) were acquired from Sigma-Aldrich (St. Louis, MO, USA). MFE-PAK^®^ SCX SPE commercial cartridges with amorphous silica functionalised with sulfonic acid as sorbent were obtained from Análisis Vínicos (Tomelloso, Spain).

Ammonia (32% (*w*/*w*)) and hydrochloric acid were received from Scharlab (Barcelona, Spain). Formic acid (99%, Optima™ LC-MS grade) was purchased from Fisher Chemical (Madrid, Spain). Methanol and acetonitrile (HPLC-MS quality) were acquired from Scharlab (Barcelona, Spain). Ultrapure water (resistivity 18.2 MΩ cm) was acquired from a Milli-Q water purification system (Millipore, Billerica, MA, USA). To prepare cartridges for SPE, nylon filter membranes (0.45 µm) and polyethylene frits (0.20 µm) were obtained from Scharlab (Barcelona, Spain).

### 4.2. Samples

Three different brands of poppy seeds (PS-01, PS-02, and PS-03) were acquired from supermarkets in Madrid (Spain) (Table S6). The packaging of these seeds indicated their suitability for various culinary uses, such as incorporation into cakes and breads, salads, creams, etc., either in whole form or after being ground. Then, each of the acquired seeds was ground using an automatic mortar (Retsch, RM 200, Germany) for 2 min, ensuring complete grinding. The ground seeds are referred to by the codes GPS-01, GPS-02, and GPS-03.

### 4.3. Synthesis of SBA-15-SO_3_^−^ Material

SBA-15 material was synthesised according to the method optimised by Zhao et al. [34]. First, 19.36 g of P123 was added to 576 mL of 2 M HCl and 144 mL of water. This mix was magnetically stirred at 35 °C in a silicon bath until complete dissolution. Later, 40.8 g of TEOS was dropwise added and magnetically stirred for 20 h. Stirring was then stopped, the temperature was increased to 80 °C, and it was left for 24 h at that temperature for an ageing period. Afterwards, the material was filtered off and washed with water. Finally, the material was dried in air and, at the end, calcined by ramping for 8.5 h to 500 °C and maintaining at 500 °C for 12 h.

SBA-15 silica functionalised with sulfonic acid was prepared according to González-Gómez et al. [26]. For this purpose, a simple synthesis route with two steps was used, which involves an initial functionalisation with thiol groups (-SH) and an oxidation afterwards to sulfonic acid groups (SO_3_^−^). First, 2.5 g of SBA-15 and 1.06 g of MPTES were added in 250 mL of 0.1 M HCl. After being magnetically stirred for 7 h at 180 rpm at room temperature, the mixture was moved to a reactor of Teflon-coated stainless steel (V 1.0 L, PS 131 bar, Parr Instrument Company, Moline, Illinois, USA) at 100 °C for 24 h. The solid was then filtered, washed with ethanol and Milli-Q water, and finally dried at 50 °C overnight. Afterwards, oxidation was carried out. For this purpose, the solid was suspended in 325 mL of 2 M HCl, and then 11.4 g of H_2_O_2_ (30%) was added. After magnetically stirring at room temperature for 5 min, the mixture was transferred to the reactor at 100 °C for 6 h. Finally, the solid was filtrated and washed with Milli-Q water and ethanol.

To confirm the successful synthesis of the material, a characterization was performed (for more information, see Supplementary Material S.1).

### 4.4. Optimisation of the Sample Preparation

The optimisation process for sample preparation involved a sequential optimisation of the SPE purification step, followed by the optimisation of UAE conditions with the design of experiments. This sequential approach was adopted to ensure the purification of all extracts during the optimisation of the UAE. By injecting cleaner extracts, the risk of equipment deterioration was minimised, enhancing the overall efficiency of the analytical process.

#### 4.4.1. Optimisation of the Purification via SPE

To carry this out, an SPE Supelco Visiprep 12-port model vacuum manifold (Sigma-Aldrich, St. Louis, MO, USA) coupled to a vacuum pump at 7.6 psi was used. Empty 3 mL polypropylene cartridges (length of 65 mm and i.d. of 10 mm) were filled with the material and plugged at both ends with polyethylene frits. Additionally, a nylon filter membrane with a 0.45 µm pore size was inserted at the underside of the material bed to avoid material loss when loading the sample.

First, a comparison was made between the synthesised material (SBA-15-SO_3_^−^) and a commercial material based on amorphous silica functionalised with sulfonic groups (MFE-PAK^®^ SCX) with different amounts of material to evaluate the efficiency of the synthesised material. For this purpose, similar conditions were used based on previous work using materials with strong acid exchange groups to analyse OAs in hot pots [23]. Therefore, 2 mL of water with 1% HCl was used to conditionate the cartridge, 2 mL of 0.5 mg/L of OAs in water with 1% HCl was used for sample loading, and 2 mL of methanol with 5% ammonia was used for elution. After elution, the purified extracts obtained were evaporated and reconstituted in water/acetonitrile 90/10 with 0.1% formic acid for subsequent analysis via HPLC-MS/MS. These assays were performed in triplicate. Subsequently, a re-optimisation of the SPE procedure was performed with the material SBA-15-SO_3_^−^. First, the recovery values obtained with the methanol elution with only 1% ammonia were evaluated. The objective was to determine if this elution medium could be employed to inject it directly without evaporation, thereby significantly reducing the overall analysis time.

Following the optimisation of SPE using standards, the cleaning step with the extract was optimised. For this purpose, the ground poppy seed sample was spiked immediately before the purification process with a known concentration (5 mg/L) to determine the recovery values for each of the analytes in that step. Subsequently, it was evaluated whether and at what ratio (undiluted, 1/10 dilution, and 1/20 dilution) the extract needed dilution to achieve acceptable recovery values. Dilutions were made in water with 1% HCl. Then, as the solvents used for the optimisation of the UAE will be water, methanol, and ethanol, it was evaluated to ensure that the SPE procedure showed adequate recovery values with each of them.

#### 4.4.2. Optimisation of UAE Using Design Experiments

A three-factor, three-level full factorial design (3^3^) was used to evaluate the solvent type (A), the solid/liquid ratio (B), and the extraction time (C) more efficiently in the recovery of OAs. A selection of the three most encouraging levels was made, as presented in Table S1. The selection of levels for each independent variable was based on previous preliminary experiments and related research [18]. Subsequently, the optimal levels for the extraction time and the solid/liquid ratio were determined for each solvent type via RSM.

### 4.5. Optimised Analysis Methodology for Quantifying Opium Alkaloids in Ground Poppy Seeds

**Figure 6:**
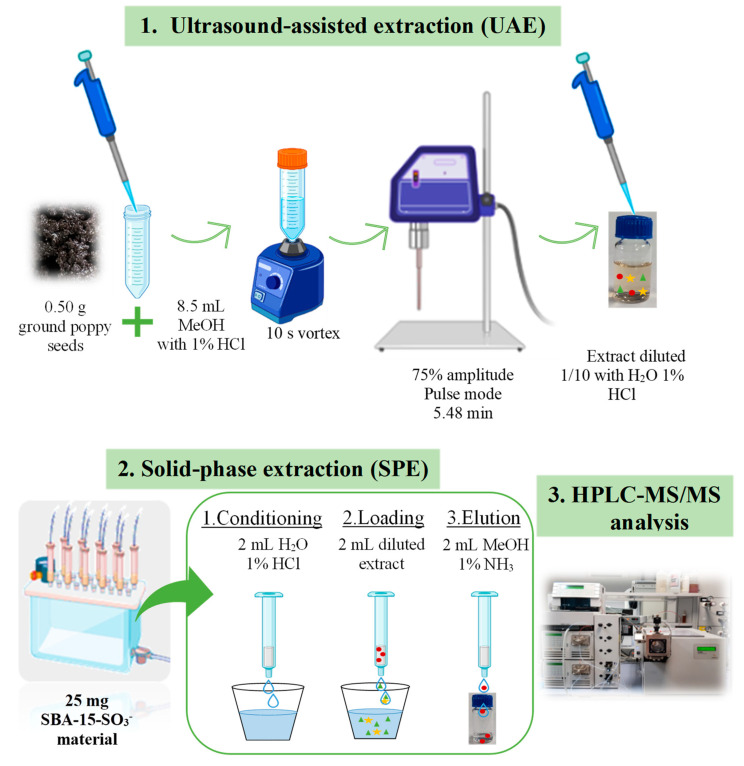
Proposed methodology diagram for the quantification of OAs in ground poppy seeds based on a UAE and an SPE for purification and analysis via HPLC-MS/MS.

The developed and optimised method was based on a UAE, following SPE with SBA-15-SO_3_^−^ material and subsequent analysis via HPLC-MS/MS, as presented in Figure 6.

First, the extraction of OAs from the ground seeds was carried out with a UAE. For this purpose, 0.5 g of sample was mixed with 8.5 mL of MeOH with 1% HCl for 10 s using a vortex (Rx^3^ Velp Scientifica, Usmate, MB, Italy). Subsequently, the mixture was subjected to acoustic waves in controlled conditions in the UAE as obtained in the experimental design using Bandelin Sonopuls 529 (Amplichron ^®^-System, Bandelin, Berlin, Germany) with an MS 73 probe with a diameter of 13 mm. The mixture, placed in a 50 mL falcon tube, was exposed to pulsed mode at 75% amplitude for 5 min and 48 s. Afterwards, it was centrifuged for 5 min at 9000 rpm (Digicen 21 R from Ortoalresa, Madrid, Spain).

Then, the extracts were purified via SPE under optimised conditions. For this purpose, 25 mg of silica SBA-15 functionalised with sulfonic groups (SBA-15-SO_3_^−^) was employed. The process included a conditioning step with 2 mL of water with 1% HCl, a loading with 2 mL of the extract (diluted 1/10 with water with 1% HCl and adjusting the pH to 1), and an elution with 2 mL of methanol with 1% ammonia. Subsequently, an aliquot of 950 µL was taken, and 50 µL of a 1 µg/mL dilution of morphine-d_3_ and codeine-d_3_ (IS) was added before analysis via HPLC-MS/MS.

For the analysis of OAs in ground poppy seeds, a Varian 1200/1200 LC (Varian Ibérica, Madrid, Spain) composed of a ProStar 410 autosampler (100 µL loop) was used. It was coupled to a tandem mass spectrometer detector with a triple-quadrupole-type analyser (1200 L TQ). The ion source used was electrospray ionisation (ESI), using the MS Workstation Varian data acquisition system (version 6.8). The chromatographic conditions were conducted following the methodology outlined in our prior research [4]. The injection volume was 10 µL via partial injection. The column used was a C18 Kromaphase 100 at 30 °C with dimensions of 150 × 2.0 mm and a particle size of 3.5 µm (Scharlab, Barcelona, Spain). The flow rate was 0.25 mL/min in a gradient elution formed with water (A) and acetonitrile (B), both with 0.1% formic acid. The method started with 90% of A and, in minute 6, changed to 30% A to change again in minute 9 to the initial conditions and maintained 2 min more to re-equilibrate the column. The mass spectrometric acquisition was conducted via electrospray ionisation in positive mode (ESI+) with multiple reaction mode (MRM), as in our previous work [4]. The drying gas was N_2_ at 22 psi and 350 °C, and the nebuliser was also N_2_ at 58 psi. The capillary voltage was 5000 V and shielding 600 V. The collision gas used was argon at 2.00 mTorr, and the detector voltage was 1553 V. The mass peak width was Q1 2.5, the mass peak width was Q3 2.5, and the MRM scan width was 0.5 s. The cone voltage for the monitored compounds was 72 V. The optimal parameters of MRM for the analysis of six opium alkaloids are shown in Table S7.

### 4.6. Method Validation

The validation of the proposed methodology to quantify opium alkaloids in ground poppy seeds was performed in terms of linearity, matrix effect (ME), method detection and quantification limits (MDL and MQL), accuracy, precision, and selectivity. Nowadays, there is no official regulation for validating analytical methods for OAs in food or feed. Therefore, the method validation in this study was carried out in the present work following the SANTE/11312/2021 document [28], regulation EC No. 401/2006 [35], and Q2(R1) ICH guidelines (International Council for Harmonisation, 2005) [36]. For the validation, the commercial poppy seed PS01 (Table S6) was used, chosen for its low levels of each OA. Additionally, a double wash with water at 100 °C for 30 min was applied, following the procedure established in our prior research on whole poppy seeds [4].

First, the linear regression analysis was evaluated with matrix-matched calibration curves made in three different days. These calibration curves were made for sample GPS-01, and six known concentration levels were evaluated between 0.001 and 1 ppm. To complete this, the GPS-01 sample was subjected to the UAE procedure and subsequent purification via SPE, and just before being analysed via HPLC-MS/MS, it was spiked with an aliquot of a standard solution containing the target alkaloids according to the desired concentration level. Furthermore, an isotope-labelled IS correction was performed for quantification. For this purpose, 50 µL of 0.1 µg/mL from each IS was spiked at each point. Additionally, according to the validation guidelines, good linearity criteria dictate that the deviation from back-calculated concentrations of calibrating standards should be within ≤±20% of actual concentrations [27,35].

Regarding the matrix effect, it was established by comparing the slopes of equations of calibration curves from matrix and solvent calibration curves. That is, calculating with the following formula: (slope matrix-matched/slope solvent-based − 1) × 100 for each analyte.

The MDLs and MQLs were calculated to assess the sensitivity of the method with respect to the OAs. To calculate each of these, the lowest concentration analysed was estimated as the concentration giving a signal-to-noise ratio (S/N) of 3 or 10, respectively [27].

To determine the accuracy of the proposed method, recovery tests were performed at three different concentration levels: 40 mg/kg (high validation level), 20 mg/kg (medium validation level), and 3.4 mg/kg (low validation level) by Regulation (EU) 2023/915, establishing maximum morphine equivalent limits in poppy seeds intended for direct human consumption at 20 mg/kg [6]. The blank sample (washed PS01) was used to determine the recovery values. To calculate this, the areas obtained for samples (*n* = 6) spiked at the corresponding concentration level and subjected to the developed sample preparation were compared with the areas obtained for simulated samples (samples spiked at the respective concentration but before analysis via HPLC-MS/MS). According to the validation guidelines, the recovery values should be between 70 and 120% [27]. In addition, the precision of the method was determined by its repeatability and reproducibility, using the same validation levels. Intra-day precision (repeatability, RSD %) was calculated six times on the same day (*n* = 6) on the same day and inter-day precision (reproducibility, RSD %) was evaluated with three replicates on three different days (*n* = 9). Following the validation guidelines, the RSD (%) values for the parameters should be ≤20% [27].

Finally, the selectivity of the method was evaluated using the spectra obtained for each of the analytes from standards compared with the spectra obtained with samples. It was considered adequate if the variation of the spectra was less than ±30% and the retention time of the target analytes was within the range of ±2.5% for each analyte [27].

### 4.7. Greenness Evaluation of the Proposed Analytical Methodology

The eco-friendly properties of the proposed analytical methodology for determining OAs in ground poppy seed samples were evaluated for greenness using the Analytical Greenness Metric for Sample Preparation (AGREEprep) [31]. This metric is based on 10 consecutive steps of assessment corresponding to the 10 principles of Green Sample Preparation [37]. Furthermore, it provides insights into the strengths and weaknesses of the procedure. Evaluation categories encompass factors such as the consumption of hazardous reagents, waste generation, sample quantity, and energy consumption, among others. For each assessed item, researchers can assign a score based on its perceived importance to the overall procedure outcome. Finally, a final pictogram is generated to summarise the ecological character of the method.

### 4.8. Statistical Analysis

Statistical analysis and design of experiments were performed using Statgraphics Centurion software (version 19.3.03). Differences were considered significant for *p*-values ≤ 0.05.

## Figures and Tables

**Table 1:** Results from the factorial experimental design for ultrasound-assisted extraction (UAE) optimisation.

RUN	Factor *			Responses (Recovery Mean (%) ± SD)					
	A	B	C	Morphine (Y_1_)	Codeine (Y_2_)	Thebaine (Y_3_)	Papaverine (Y_4_)	Noscapine (Y_5_)	Oripavine (Y_6_)
1	1	−1	−1	10 ± 2	17 ± 2	24 ± 3	28 ± 2	18 ± 1	51 ± 1
2	1	−1	0	17 ± 1	34 ± 1	32 ± 2	33 ± 3	29 ± 2	53 ± 2
3	1	−1	1	29 ± 3	42 ± 3	42 ± 3	40 ± 2	38 ± 4	43 ± 4
4	1	0	−1	28 ± 2	48 ± 4	52 ± 2	58 ± 1	49 ± 2	60 ± 1
5	1	0	0	28 ± 1	49 ± 2	53 ± 1	59 ± 2	53 ± 1	69 ± 2
6	1	0	1	37 ± 1	60 ± 3	60 ± 3	71 ± 2	62 ± 2	72 ± 3
7	1	1	−1	27 ± 3	40 ± 2	43 ± 2	47 ± 1	39 ± 3	76 ± 4
8	1	1	0	39 ± 2	69 ± 1	72 ± 4	81 ± 2	65 ± 2	76 ± 5
9	1	1	1	45 ± 1	85 ± 2	88 ± 2	91 ± 1	67 ± 2	26 ± 1
10	2	−1	−1	42 ± 1	40 ± 3	28 ± 1	5 ± 3	40 ± 1	37 ± 2
11	2	−1	0	46 ± 3	50 ± 2	30 ± 2	7 ± 2	46 ± 3	47 ± 2
12	2	−1	1	50 ± 1	55 ± 4	36 ± 3	7 ± 1	52 ± 1	46 ± 1
13	2	0	−1	52 ± 2	57 ± 1	38 ± 2	8 ± 2	52 ± 2	75 ± 6
14	2	0	0	62 ± 3	59 ± 3	38 ± 2	8 ± 3	55 ± 3	69 ± 5
15	2	0	1	64 ± 1	61 ± 2	39 ± 1	11 ± 2	56 ± 1	77 ± 4
16	2	1	−1	67 ± 5	72 ± 1	43 ± 4	12 ± 1	62 ± 2	73 ± 3
17	2	1	0	69 ± 2	73 ± 3	44 ± 2	13 ± 2	63 ± 1	54 ± 2
18	2	1	1	72 ± 3	73 ± 2	45 ± 4	13 ± 1	63 ± 2	57 ± 1
19	3	−1	−1	45 ± 2	48 ± 3	40 ± 2	58 ± 4	48 ± 4	54 ± 3
20	3	−1	0	49 ± 2	52 ± 2	47 ± 3	63 ± 2	55 ± 2	62 ± 2
21	3	−1	1	53 ± 3	57 ± 1	48 ± 2	65 ± 3	60 ± 4	65 ± 3
22	3	0	−1	54 ± 4	86 ± 3	72 ± 3	74 ± 4	75 ± 1	66 ± 2
23	3	0	0	81 ± 1	100 ± 2	94 ± 2	92 ± 5	97 ± 2	78 ± 3
24	3	0	1	81 ± 2	100 ± 4	97 ± 4	93 ± 3	100 ± 5	82 ± 4
25	3	1	−1	68 ± 3	82 ± 2	73 ± 2	80 ± 5	72 ± 2	39 ± 3
26	3	1	0	80 ± 2	100 ± 2	96 ± 3	97 ± 2	98 ± 3	45 ± 2
27	3	1	1	82 ± 1	100 ± 4	99 ± 1	100 ± 4	100 ± 1	55 ± 1

SD: standard deviation (*n =* 3). * (A) solvent type, (B) solid/liquid ratio, and (C) extraction time (min) to UAE optimisation.

**Table 2:** Validation parameters of the UAE-SPE-HPLC-MS/MS method for the quantification of six opium alkaloids in ground poppy seeds.

Analytes	Linear Range (mg/L)	Matrix-Matched Calibration (R^2^)	MDL (mg/kg) ^a^	MQL (mg/kg) ^b^	ME ^c^	Accuracy ^d^		Precision	
						Recovery (% ± SD)	Mean Recovery (% ± SD)	Intra-Day Precision (RSD %)	Inter-Day Precision (RSD %)
Morphine	0.001–1	y = 7.02 × 10^7^x + 1.68 × 10^6^ (0.995)	0.06	0.2	9	99 ± 8 ^LL^	85 ± 6	6 ^LL^	8 ^LL^
						82 ± 6 ^ML^		3 ^ML^	7 ^ML^
						73 ± 4 ^HL^		3 ^HL^	6 ^HL^
Codeine	0.001–1	y = 7.65 × 10^7^x + 9.46 × 10^5^ (0.996)	0.03	0.1	−1	95 ± 9 ^LL^	100 ± 11	3 ^LL^	10 ^LL^
						106 ± 15 ^ML^		4 ^ML^	14 ^ML^
						101 ± 10 ^HL^		4 ^HL^	10 ^HL^
Thebaine	0.001–1	y = 6.85 × 10^8^x + 1.49 × 10^7^ (0.994)	0.01	0.05	−11	89 ± 11 ^LL^	96 ± 11	3 ^LL^	12 ^LL^
						102 ± 12 ^ML^		11 ^ML^	11 ^ML^
						98 ± 10 ^HL^		7 ^HL^	11 ^HL^
Papaverine	0.001–1	y = 1.77 × 10^9^x + 1.51 × 10^7^ (0.995)	0.01	0.05	5	97 ± 15 ^LL^	99 ± 12	12 ^LL^	15 ^LL^
						99 ± 7 ^ML^		6 ^ML^	15 ^ML^
						99 ± 6 ^HL^		3 ^HL^	7 ^HL^
Noscapine	0.001–1	y = 2.76 × 10^9^x + 4.87 × 10^7^ (0.995)	0.007	0.03	5	98 ± 9 ^LL^	94 ± 11	6 ^LL^	9 ^LL^
						92 ± 13 ^ML^		5 ^ML^	13 ^ML^
						92 ± 12 ^HL^		6 ^HL^	13 ^HL^
Oripavine	0.005–1	y = 7.60 × 10^7^x + 1.55 × 10^6^ (0.996)	0.2	0.5	−4	95 ± 11 ^LL^	94 ± 10	6 ^LL^	12 ^LL^
						95 ± 10 ^ML^		3 ^ML^	10 ^ML^
						91 ± 10 ^HL^		7 ^HL^	11 ^HL^

The linear range expressed in mg/kg is 0.17–170 for all analytes except for oripavine, that is, 0.85–170; ^a^ MDL: method detection limit; ^b^ MQL: method quantification limit; ^c^ ME: matrix effect (purified matrix slope/the solvent slope − 1) × 100; and ^d^ accuracy and precision were obtained by spiking samples at three concentration levels: low (LL, 3.4 mg/kg), medium (ML, 20 mg/kg), and high (HL, 40 mg/kg).

**Table 3:** Occurrence (mg/kg) ± SD (standard deviation) of each of the six opium alkaloids analysed in six replicates (n = 6) for three different samples of ground poppy seeds.

Sample Code	Morphine	Codeine	Thebaine	Papaverine	Noscapine	Oripavine
GPS-01	0.8 ± 0.2	0.5 ± 0.1	0.06 ± 0.01	˂MQL	0.04 ± 0.01	˂MQL
GPS-02	4.2 ± 0.6	1.4 ± 0.4	0.7 ± 0.2	1.2 ± 0.4	3.7 ± 1.1	ND
GPS-03	134.6 ± 1.6	28.2 ± 0.1	75 ± 1	ND	0.9 ± 0.1	82.1 ± 6.3

GPS: ground poppy seeds; ND: not detected; and ˂MQL: lower than the method quantification limit but higher than the method detection limit (MDL). These seeds were purchased whole (Table S6) and then ground in an automatic mortar (Retsch, RM 200, Haan Germany) for 2 min.

## Supplementary Materials

The following supporting information can be downloaded at https://www.mdpi.com/article/10.3390/toxins15120672/s1, Table S1: summary of the three factors studied with their codes and the levels of independent variables used in three-level full factorial screening; Table S2: analysis of variance (ANOVA) report for the model; Table S3: adjusted model equation of each type of variable by response surface model; Table S4: comparison of the proposed analytical methodology for determination of six opium alkaloids from ground poppy seeds; Table S5: input used to assign AGREEprep scores of the three methods compared; S.1: characterization of SBA-15-SO_3_^−^ material; Table S6: commercial information on the different poppy seed samples analysed; and Table S7: optimal parameters of multiple reaction modes for the analysis of six opium alkaloids via HPLC-MS/MS.

## References

[1] Ghafoor K., Özcan M.M., AL-Juhaimi F., Babiker E.E., Fadimu G.J. (2019). Changes in quality, bioactive compounds, fatty acids, tocopherols, and phenolic composition in oven- and microwave-roasted poppy seeds and oil. LWT.

[2] Casado-Hidalgo G., Morante-Zarcero S., Pérez-Quintanilla D., Sierra I. (2021). Opium alkaloids in food products: Current and future perspectives. Trends Food Sci. Technol..

[3] AESAN (Spanish Food Safety and Nutrition Agency) (2020). Opium Alkaloids in Poppy Seeds. http://www.aecosan.msssi.gob.es/AECOSAN/docs/documentos/seguridad_alimentaria/gestion_riesgos/opio_semillas_adormidera.pdf.

[4] Casado-Hidalgo G., Pérez-Quintanilla D., Morante-Zarcero S., Sierra I. (2021). Mesostructured Silica-Coated Magnetic Nanoparticles to Extract Six Opium Alkaloids in Poppy Seeds Prior to Ultra-High-Performance Liquid Chromatography-Tandem Mass Spectrometry Analysis. Foods.

[5] EFSA (2018). Update of the Scientific Opinion on opium alkaloids in poppy seeds. EFSA J..

[6] Commission Regulation (2023). (EU) 2023/915 of 25 April 2023 on maximum levels for certain contaminants in food and repealing Regulation (EC) No 1881/2006. Off. J. Eur. Union.

[7] BfR, German Federal Institute for Risk Assessment (2006). BfR recommends provisional daily upper intake level and a guidance value for morphine in poppy seeds. BfR Health Assess..

[8] European Commission (2014). Commission Recommendation 2014/662/EU of 10 September 2014 on good practices to prevent and to reduce the presence of opium alkaloids in poppy seeds and poppy seed products. Off. J. Eur. Union.

[9] Sproll C., Perz R.C., Buschmann R., Lachenmeier D.W. (2007). Guidelines for reduction of morphine in poppy seed intended for food purposes. Eur. Food Res. Technol..

[10] Casado N., Casado-Hidalgo G., González-Gómez L., Morante-Zarcero S., Sierra I. (2023). Insight into the Impact of Food Processing and Culinary Preparations on the Stability and Content of Plant Alkaloids Considered as Natural Food Contaminants. Appl. Sci..

[11] Kanu B. (2021). Recent developments in sample preparation techniques combined with high-performance liquid chromatography: A critical review. J. Chromatogr. A.

[12] Ballesteros-Vivas D., Socas-Rodríguez B., Mendiola J.A., Ibáñez E., Cifuentes A. (2021). Green food analysis: Current trends and perspectives. Curr. Opin. Green Sustain. Chem..

[13] Câmara J.S., Perestrelo R., Berenguer C.V., Andrade C.F.P., Gomes T.M., Olayanju B., Kabir A., Rocha C.M.R., Teixeira J.A., Pereira J.A.M. (2022). Green Extraction Techniques as Advanced Sample Preparation Approaches in Biological, Food, and Environmental Matrices: A Review. Molecules.

[14] Albero B., Tadeo J.L., Pérez R.A. (2019). Ultrasound-assisted extraction of organic contaminants. Trends Anal. Chem..

[15] Tiwari B.K. (2015). Ultrasound: A clean, green extraction technology. Trends Anal. Chem..

[16] Baş D., Boyacı İ.H. (2007). Modeling and optimization I: Usability of response surface methodology. J. Food Eng..

[17] Bezerra M.A., Santelli R.E., Oliveira E.P., Villar L.S., Escaleira L.A. (2008). Response surface methodology (RSM) as a tool for optimization in analytical chemistry. Talanta.

[18] Casado-Hidalgo G., Morante-Zarcero S., Pérez-Quintanilla D., Sierra I. (2022). Pulsed ultrasound-assisted extraction followed by purification with SBA-15 for the control of opium alkaloids in biscuits and sponge cakes. Microchem. J..

[19] Andrade-Eiroa A., Canle M., Leroy-Cancellieri V., Cerdà V. (2016). Solid-phase extraction of organic compounds: A critical review (Part I). Trends Anal. Chem..

[20] Ötles S., Kartal C. (2016). Solid-Phase Extraction (SPE): Principles and Applications in Food Samples. Acta Sci. Pol. Technol. Aliment..

[21] Hayes L.W., Krasselt W.G. (1987). Concentrations of Morphine and Codeine in Serum and Urine after Ingestion of Poppy Seeds. Clin. Chem..

[22] Özbunar E., Aydoğdu M., Döğer R., Bostancı H.İ., Koruyucu M., Akgür S.A. (2019). Morphine Concentrations in Human Urine Following Poppy Seed Paste Consumption. Forensic Sci. Int..

[23] Guo Q., Zhang J., Zhao S., Shao B. (2013). Determination of Five Alkaloids of Pericarpium Papaveris in Hot Pot Broth Using Ultra-Performance Liquid Chromatography Coupled to Triple Quadruple Mass Spectrometry. Food Anal. Methods.

[24] Maciel E.V.S., de Toffoli A.L., Neto E.S., Nazario C.E.D., Lanças F.M. (2019). New materials in sample preparation: Recent advances and future trends. Trends Anal. Chem..

[25] Wang D., Chen X., Feng J., Sun M. (2022). Recent advances of ordered mesoporous silica materials for solid-phase extraction. J. Chromatogr. A.

[26] González-Gómez L., Gañán J., Morante-Zarcero S., Pérez-Quintanilla D., Sierra I. (2020). Sulfonic Acid-Functionalized SBA-15 as Strong Cation-Exchange Sorbent for Solid-Phase Extraction of Atropine and Scopolamine in Gluten-Free Grains and Flours. Foods.

[27] European Union (2021). Analytical Quality Control and Method Validation Procedures for Pesticide Residues Analysis in Food and Feed.

[28] López P., Fauw D.P.K.H.P.-D., Mulder P.P.J., Spanjer M., de Stoppelaar J., Mol H.G.J., de Nijs M. (2018). Straightforward analytical method to determine opium alkaloids in poppy seeds and bakery products. Food Chem..

[29] Sproll C., Perz R.C., Lachenmeier D.W. (2006). Optimized LC/MS/MS Analysis of Morphine and Codeine in Poppy Seed and Evaluation of Their Fate during Food Processing as a Basis for Risk Analysis. J. Agric. Food Chem..

[30] Carlin M.G., Dean J.R., Ames J.M. (2020). Opium Alkaloids in Harvested and Thermally Processed Poppy Seeds. Front. Chem..

[31] Pena-Pereira F., Tobiszewski M., Wojnowski W., Psillakis E.A. (2022). Tutorial on AGREEprep an Analytical Greenness Metric for Sample Preparation. Adv. Sample Prep..

[32] EURL-MP-Method_007. Determination of Opium Alkaloids in Poppy Seeds and Poppy Seed Containing Bakery Products by LC-MS/MS. https://www.wur.nl/en/research-results/research-institutes/food-safety-research/reference-laboratory/european-union-reference-laboratory/eurl-mycotoxins-plant-toxins.htm.

[33] Powers D., Erickson S., Swortwood M.J. (2018). Quantification of Morphine, Codeine, and Thebaine in Home-Brewed Poppy Seed Tea by LC-MS/MS. J. Forensic Sci..

[34] Zhao D., Huo Q., Feng J., Chmelka B.F., Stucky G.D. (1998). Nonionic Triblock and Star Diblock Copolymer and Oligomeric Surfactant Syntheses of Highly Ordered, Hydrothermally Stable, Mesoporous Silica Structures. J. Am. Chem. Soc..

[35] (2006). European Commission Regulation No. 401/2006 of 23 February 2006 Laying down the Methods of Sampling and Analysis for the Official Control of the Levels of Mycotoxins in Foodstuffs. https://eur-lex.europa.eu/legal-content/EN/TXT/PDF/?uri=CELEX:32006R0401&from=EN.

[36] Tietje C., Brouder A. (2010). Q2(R1) ICH guidelines (Internation Council for Harmonisation, 2005). Handbook of Transnational Economic Governance Regimes.

[37] López-Lorente Á.I., Pena-Pereira F., Pedersen-Bjergaard S., Zuin V.G., Ozkan S.A., Psillakis E. (2022). The ten principles of green sample preparation. Trends Anal. Chem..

