# Supplementary material for: Design and Optimisation of Sustainable Sample Treatments Based on Ultrasound-Assisted Extraction and Strong Cation-Exchange Purification with Functionalised SBA-15 for Opium Alkaloids in Ground Poppy Seeds
Source: Toxins (Basel). 2023 Nov 24;15(12):672. doi: 10.3390/toxins15120672 (PMC10747185; doi:10.3390/toxins15120672)
Supplement: Supplementary file 1 [file toxins-15-00672-s001.zip › toxins-2601853-supplementary.pdf]

# Design and optimization of sustainable sample treatment based on ultrasound-assisted extraction and strong cation-exchange purification with functionalized SBA-15 for opium alkaloids in ground poppy seeds

**Gema Casado-Hidalgo, Sonia Morante-Zarcero, Damián Pérez-Quintanilla, Isabel Sierra**

**Table S1.** Summary of the 3 factors studied with their codes and the levels of independent variables used in three-level full factorial screening.

| Factor type | Symbol | Factor levels | | |
|---|---|---|---|---|
| - **Factor categorical** | | **Type 1** | **Type 2** | **Type 3** |
| Solvent type | A | Ethanol | Water | Methanol |
| - **Factor numerical** | | **Low (1)** | **Medium (0)** | **High (+1)** |
| Solid-liquid ratio | B | 0.5g/3mL | 0.5g/5mL | 0.5g/10mL |
| Extraction time (min) | C | 3 | 5 | 10 |

**Table S2.** Analysis of variance (ANOVA) report for the model.

| Variable | Morphine recovery (%) | Codeine recovery (%) | Thebaine recovery (%) | Papaverine recovery (%) | Noscapine recovery (%) | Oripavine recovery (%) |
|---|---|---|---|---|---|---|
| **$R^2$** | 0.9647 | 0.9060 | 0.9110 | 0.9742 | 0.9360 | 0.9222 |
| **$R^2$ Adj.** | 0.9388 | 0.8370 | 0.8458 | 0.9553 | 0.8891 | 0.8652 |
| **$R^2$ Pred.** | 0.9012 | 0.6842 | 0.6878 | 0.9124 | 0.7679 | 0.7415 |
| | | | **p-value** | | | |
| **A** | 0.0006* | 0.0146* | 0.2048 | 0.0017* | 0.0307* | 0.0429* |
| **B** | 0.0001* | 0.0003* | 0.0006* | 0.0026* | 0.0016* | 0.001* |
| **C** | 0.0120* | 0.0863 | 0.1333 | 0.0713 | 0.0889 | 0.0218* |
| **AB** | 0.4416 | 0.6007 | 0.0668 | 0.0038* | 0.0325* | 0.3387 |
| **AC** | 0.4006 | 0.1761 | 0.2144 | 0.0876 | 0.0828 | 0.2416 |
| **BB** | 0.0004* | 0.0009* | 0.0012* | 0.0035* | 0.0028* | 0.0002* |
| **BC** | 0.8551 | 0.5693 | 0.2544 | 0.0640 | 0.2510 | 0.7642 |
| **CC** | 0.0361* | 0.1236 | 0.1470 | 0.0558 | 0.0974 | 0.0395* |

*significant $p < 0.05$.

**(1)**

**Table S3.** Adjusted model equation of each type of variable by response surface model.

| Variable | Adjusted model equation |
| --- | --- |
| $Y_1$ | $Y_1 = 55.4 + 7.2 \times A_{[1]} + 14.9 \times A_{[2]} + 11.6 \times B + 6.7 \times C + 0.1 \times A_{[1]}B + 2.3 \times A_{[2]}B - 2.5 \times A_{[1]}C + 1.5 \times A_{[2]}C - 4.7 \times B^2 + 0.1 \times BC - 2.0 \times C^2$ |
| $Y_2$ | $Y_2 = 70.7 - 3.3 \times A_{[1]} + 17.3 \times A_{[2]} + 16.6 \times B + 7.9 \times C - 4.4 \times A_{[1]}B + 4.2 \times A_{[2]}B - 4.6 \times A_{[1]}C - 1.1 \times A_{[2]}C - 8.4 \times B^2 + 1.3 \times BC - 2.7 \times C^2$ |
| $Y_3$ | $Y_3 = 62.0 - 16.7 \times A_{[1]} + 19.4 \times A_{[2]} + 15.3 \times B + 7.8 \times C - 9.0 \times A_{[1]}B + 6.8 \times A_{[2]}B - 6.0 \times A_{[1]}C + 2.0 \times A_{[2]}C - 8.7 \times B^2 + 3.3 \times BC - 2.5 \times C^2$ |
| $Y_4$ | $Y_4 = 53.9 - 41.0 \times A_{[1]} + 32.3 \times A_{[2]} + 12.1 \times B + 6.7 \times C - 10.9 \times A_{[1]}B + 3.1 \times A_{[2]}B - 5.6 \times A_{[1]}C + 0.9 \times A_{[2]}C - 5.5 \times B^2 + 4.5 \times BC - 3.5 \times C^2$ |
| $Y_5$ | $Y_5 = 65.2 - 26.6 \times A_{[1]} + 24.0 \times A_{[2]} + 14.3 \times B + 8.0 \times C - 9.9 \times A_{[1]}B + 3.2 \times A_{[2]}B - 6.8 \times A_{[1]}C + 0.3 \times A_{[2]}C - 7.5 \times B^2 + 3.6 \times BC - 3.3 \times C^2$ |
| $Y_6$ | $Y_6 = 69.1 - 5.4 \times A_{[1]} + 18.6 \times A_{[2]} + 13.5 \times B + 7.9 \times C - 5.2 \times A_{[1]}B + 4.3 \times A_{[2]}B - 5.1 \times A_{[1]}C + 2.9 \times A_{[2]}C - 10.2 \times B^2 + 1.1 \times BC - 3.8 \times C^2$ |

$Y_1$: morphine recovery (%); $Y_2$: codeine recovery (%); $Y_3$: thebaine recovery (%); $Y_4$: papaverine recovery (%); $Y_5$: noscapine recovery (%); $Y_6$: oripavine recovery (%); A: solvent type; B: solid-liquid ratio; C: extraction time.

**Table S4.** Comparison of the proposed analytical methodology for determination of six opium alkaloids from ground poppy seeds.

| Sample (amount) | Sample treatment Extraction | Purification | Analysis technique | Matrix effect | Recovery (%) | RSD (%) | Refs. |
|---|---|---|---|---|---|---|---|
| **Unground poppy seeds (10 g)** | **MeOH 0.1% acetic acid (30 mL, 60 min)** | **-** | **HPLC-TQ-MS/MS** | **-** | - | ≤9 | [41] |
| Unground poppy seeds (10 g) | AcN/water/formic acid, 80/19/1, v/v/v (100 mL, 30 min x 2) | - | UHPLC-TQ-MS/MS | 20-50 | 77-172 | ≤20 | [40] |
| Unground poppy seeds (0.2 g) | Chl/IPOH (90/10, v/v) at pH 3.5 (1 mL, 10 min) | - | HPLC-IT-MS/MS | - | - | ≤6 | [42] |
| Unground poppy seeds (2.5 g) | MeOH/water, 50/50, v/v (30 mL, 30 min x 2) | MSPE: $Fe_3O_4$@$SiO_2$@m$SiO_2$ (50 mg) | UHPLC-TQ-MS/MS | -39-29 | 46-109 | ≤11 | [2] |
| Ground poppy seeds (0.5 g) | MeOH 1% HCl (8.5 mL, 5.5 min) | SPE: SBA-15-$SO_3^-$ (25 mg) | HPLC-TQ-MS/MS | -11-9 | 85-100 | ≤15 | This work |

MeOH: methanol; AcN: acetonitrile; Chl: chloroform; IPOH: isopropanol; HCl: hydrochloride acid; (M)SPE: (magnetic) solid phase extraction; (U)HPLC: (ultra)-high-performance liquid chromatography; IT: ion trap; TQ: triple quadrupole; MS/MS: tandem mass spectrometry; RSD: relative standard deviation; Refs.: references.

**Table S5.** Input used to assign AGREEprep scores of the three methods compared.

| Item | Weight | The proposed methodology in this work (UAE-SPE-HPLC-MS/MS) | | Example 1 (SLE-MSPE-HPLC-MS/MS) [2] | | Example 2 (SLE-HPLC-MS/MS) [41] | |
|---|---|---|---|---|---|---|---|
| | | **Input** | **Justification for input** | **Input** | **Justification for input** | **Input** | **Justification for input** |
| 1 | 1 | Ex situ | Sample preparation performed in the lab | Ex situ | Sample preparation performed in the lab | Ex situ | Sample preparation performed in the lab |
| 2 | 5 | 10.635 | 8.5 mL MeOH to UAE + 2 mL MeOH to elution in SPE + 0.115 mL HCl + 0.02 mL $NH_3$ | 34 | 30 mL MeOH to SLE + 4 mL diethyl ether/MeOH | 30.06 | 30 mL MeOH to SLE + 0.06 mL acetic acid |
| 3 | 5 | Not sustainable but are used several times. | This is because SPE cartridges can be reused 4 times. | < 25% | Nothing can be reused, and they are not sustainable. | < 25% | Nothing can be reused, and they are not sustainable. |
| 4 | 3 | 11.135 | Sum of sample and added reagents. | 67.5 | 60 mL MeOH/$H_2O$ to SLE + 2.5 g sample + 4 mL diethyl ether/MeOH + 1 mL to reconstitute. | 40.06 | Sum of sample and added reagents. |
| 5 | 5 | 0.5 | Ground poppy seed amount. | 2.5 | Poppy seed amount. | 10 | Poppy seed amount. |
| 6 | 5 | 12 | Extraction time are 5 min, so 12 samples can be prepared in one hour. | 1 | Extraction time are 60 min. | 1 | Extraction time are 60 min. |
| 7 | 1 | 3 steps | The first step is UAE, the second is centrifugation and the last is SPE. | 4 steps | The first step is SLE, the second is centrifugation, the third is MSPE and the last, evaporation | 1 step | SLE single step |
| 8 | 5 | 20.89 Wh | 135 W vortex (10 s) + 40 W ultrasound probe (5 min) + 900 W centrifuge (5 min) + 100 W vacuum bomb (5 min) | 147.74 Wh | 135 W vortex (10 s) + 50 W stirring (60 min) + 40 W US (3 min) + 900 W centrifuge (5 min) + 100 W vacuum bomb (10 min) | 50 Wh | 50 W magnetic stirring (60 min) |
| 9 | 1 | HPLC-MS/MS | Equipment used: HPLC-MS/MS | HPLC-MS/MS | Equipment used: HPLC-MS/MS | HPLC-MS/MS | Equipment used: HPLC-MS/MS |
| 10 | 1 | 3 hazards | 3 pictograms: toxic, flammable, carcinogen | 3 hazards | 3 pictograms: toxic, flammable, carcinogen | 3 hazards | 3 pictograms: toxic, flammable, carcinogen |

(a) (b) (c)

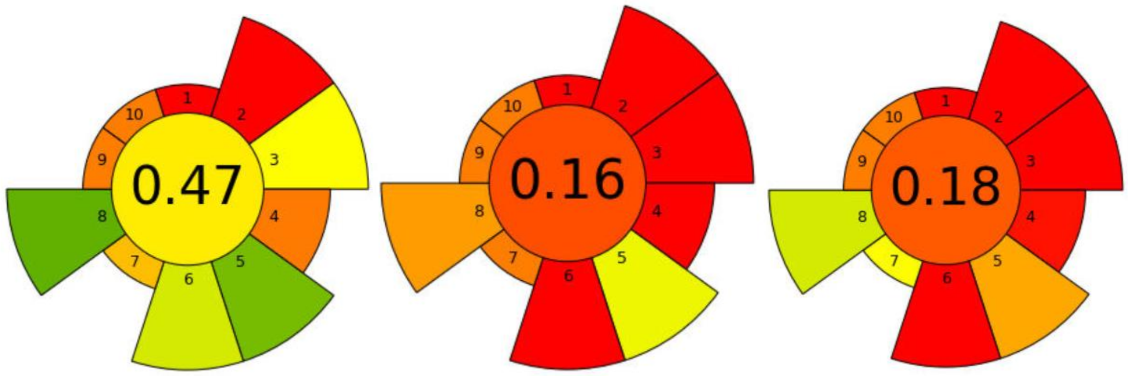


**Figure S1.** Evaluation of the greener profile of the proposed methodology in this work based on UAE-SPE-LC-MS/MS **(a)** a method based on SLE-MSPE-LC-MS/MS [2] **(b)** and a method based on SLE-LC-MS/MS without purification step [41] **(c)** using the AGREEprep metric proposed by Pena-Pereira, Tobiszewski, Wojnowski and Psillakis [38].

**Supplementary Information S.1** Characterization of SBA-15-$SO_3^-$ material

Elemental analysis (% N) was performed using a microanalyser Flash 2000 Thermo Fisher Scientific Inc. (Hampton, NH, USA) to determine the degree of functionalisation obtained and to verify that the SH- groups had been oxidised to $SO_3^-$. Nitrogen gas adsorption–desorption isotherms were obtained using a Micromeritics ASAP 2020 analyser. These isotherms were measured at -196 °C over the interval of relative pressures (P/Po) from $10^{-4}$ to 0.994. Before measurements, the samples were degassed in a vacuum at 80 °C for 10 h in the degasification unit of the instrument. These temperatures were chosen to avoid any degradation of the organics groups and to remove adsorbed species, solvents, and water. The Brunauer–Emmett–Teller (BET) method was employed to calculate the specific surface areas ($S_{BET}$). By using the Barrett–Joyner–Halenda (BJH) model, the pore volumes and pore size distributions were derived from the desorption branches of isotherms, and the total pore volumes (Vt) estimated from the desorbed amount at a relative pressure P/Po of 0.97.

**Table S6.** Commercial information on the different poppy seed samples analysed.

| Code | Description | Best-before date | Cultivation type | Origin | Recommendations for use |
|---|---|---|---|---|---|
| PS01 | Blue poppy seeds | 04/2022 | Ecological cultivation | No European Union | For the preparation of infusions |
| PS02 | Blue poppy seeds | 02/2024 | Ecological cultivation | Turkey | They can be used in juices, soups, smoothies and yoghurts. Also ground |
| PS03 | Blue poppy seeds | 07/2024 | No ecological cultivation | Unknown | Not specified |

PS: poppy seeds.

**Table S7.** Optimal parameters of multiple reaction mode for the analysis of six opium alkaloids by HPLC-MS/MS.

| Analytes | $t_R$[a] (min) | Ionization mode | Precursor ion ($Q_1$, m/z, $[M+H]^+$) | Fragment ion[b] ($Q_3$, m/z) | CE[c] (eV) |
|---|---|---|---|---|---|
| Morphine | 5.179 | ESI (+) | 286.1 | **153.0** | 45 |
| | | | | 165.0 | 24 |
| | | | | 228.6 | 22 |
| Morphine-d3 | 5.819 | ESI (+) | 288.7 | **152.3** | 45 |
| | | | | 164.2 | 37 |
| | | | | 200.6 | 25 |
| Codeine | 5.528 | ESI (+) | 300.2 | 153.1 | 45 |
| | | | | 165.0 | 45 |
| | | | | **215.1** | 23 |
| Codeine-d3 | 5.533 | ESI (+) | 303.4 | 182.2 | 30 |
| | | | | 199.0 | 30 |
| | | | | 215.1 | 24 |
| Oripavine | 5.648 | ESI (+) | 298.3 | 236.9 | 14 |
| | | | | **249.1** | 17 |
| | | | | 267.1 | 12 |
| Thebaine | 6.292 | ESI (+) | 312.3 | **58.2** | 8 |
| | | | | 166.2 | 16 |
| | | | | 249.4 | 16 |
| Papaverine | 6.507 | ESI (+) | 340.2 | **202.0** | 24 |
| | | | | 324.1 | 30 |
| Noscapine | 6.554 | ESI (+) | 414.3 | 205.1 | 24 |
| | | | | **220.0** | 42 |
| | | | | 280.1 | 20 |

[a] $t_R$: retention time; [b]: the fragment ions used for the quantification are in bold. [c] CE: collision energy.
